# The Novel lncRNA RP9P Promotes Colorectal Cancer Progression by Modulating miR-133a-3p/FOXQ1 Axis

**DOI:** 10.3389/fonc.2022.843064

**Published:** 2022-05-05

**Authors:** Zhichao Jin, Baoxinzi Liu, Bofan Lin, Ran Yang, Cunen Wu, Weiwei Xue, Xi Zou, Jun Qian

**Affiliations:** ^1^ Department of Oncology, Jiangsu Province Hospital of Chinese Medicine, Affiliated Hospital of Nanjing University of Chinese Medicine, Nanjing, China; ^2^ Jiangsu Collaborative Innovation Center of TCM Cancer Prevention and Treatment, Nanjing, China

**Keywords:** colorectal cancer, RP9P, miR-133a-3p, ceRNA, lncRNA

## Abstract

**Background:**

The long non-coding RNA (lncRNA) RP9 pseudogene (RP9P) is a pseudogene-derived lncRNA that has never been reported in cancer, and its function underlying tumorigenesis in colorectal cancer (CRC) remains unknown.

**Methods:**

RP9P and miR-133a-3p were filtered through bioinformatics analysis. The level of RP9P, miR-133a-3p, and FOXQ1 in CRC cell lines was detected by real-time PCR. Cell Counting Kit-8 and flow cytometric analyses were used to detect cell proliferation and apoptosis, respectively. Interactions between RP9P, miR-133a-3p, and FOXQ1 were confirmed by a dual-luciferase reporter assay.

**Results:**

RP9P was overexpressed in CRC compared to normal control tissues and cells. Knockdown of RP9P inhibited CRC cell viability. RP9P directly interacted with miR-133a-3p, and miR-133a-3p downregulation abrogated the tumor-suppressing effect of RP9P knockdown. miR-133a-3p directly targeted FOXQ, which was positively regulated by RP9P. RP9P knockdown decreased FOXQ1 expression levels in CRC cells by directly targeting miR-133a-3p *via* a sponge mechanism. In addition, *in vivo* experiments in a xenograft model revealed that downregulated RP9P expression inhibited CRC cell tumorigenesis.

**Conclusion:**

RP9P promotes colorectal cancer progression by regulating the miR-133a-3p/FOXQ1 axis.

## Introduction

Colorectal cancer (CRC) is one of the most common cancers in the United States, with 147,950 new cases and 53,200 deaths in 2020 (1). Although there has been great progress in CRC treatments, including surgery, chemotherapy, radiotherapy, anti-EGFR therapy, anti-VEGF, and immunotherapy, patients with CRC exhibit poor survival times (2). Thus, it is urgent to discover new therapeutic targets in CRC.

Long non-coding RNAs (lncRNAs) are a class of RNAs with non-coding domains comprising more than 200 nucleotides. Increasing evidence indicates that lncRNAs are widely implicated in cancer initiation and progression as oncogenic or tumor suppressor genes by interacting with DNA and RNA (3). Competing endogenous RNAs (ceRNAs), also called endogenous microRNA (miRNA) sponges, are one of the most common mechanisms in lncRNAs (4). The ceRNAs hypothesis stems from the studies of miRNAs, which usually block translation and accelerate degradation of target mRNAs by binding to miRNA recognition elements (MRE) and forming an RNA-induced silencing complex (RISC). The transcripts including lncRNAs, transcribed pseudogenes, circular RNAs and mRNAs can competitively bind to same MRE and affect the regulation of miRNAs on target mRNAs, thereby forming a complex RNA regulatory network (5, 6). An accumulating number of lncRNAs have been found to support this mechanism, such as the HAND2‐AS1/miR‐340‐5p/BCL2L11 axis in ovarian cancer (7) and the RPL34‐AS1/miR‐3663‐3p/RGS4 axis in papillary thyroid cancer (8). Pseudogenes are a special group of non-coding genes that have a similar DNA segment to the homologous coding gene. It has been recently recognized that pseudogenes can be transcribed into lncRNAs and interact with coding genes by competing for the same miRNA (9), such as PTENP1 (10) and DUXAP8 (11). The lncRNA RP9 pseudogene (RP9P) is a novel pseudogene-derived lncRNA located at chromosome 7:32,916,815-32,943,176.

Here, we report the first investigation of the role of RP9P in CRC development. In this study, we explored the role of the RP9P/miR-133a-3p/FOXQ1 axis in CRC. Our data suggest that RP9P serves as an oncogenic lncRNA. Furthermore, a high level of RP9P predicts worse survival in CRC. *In vitro*, loss-of-function experiments on lncRNAs indicated that RP9P played pro-tumorigenic roles in CRC by sponging hsa-miR-133a-3p/FOXQ1. In summary, these results provide neoteric insights into the treatment and diagnosis of CRC.

## Materials and Methods

### Bioinformatics Analyses

Data for bioinformatics analyses were obtained from The Cancer Genome Atlas (TCGA) database downloaded from the Xena website (https://xenabrowser.net/datapages/). TCGA-COADREAD data were selected that included 383 primary CRC and 51 non-cancer tissues. All data were normalized using R 3.5.1 with the limma package. The GTF gene annotation file (Gene transfer format) downloaded from Ensembl (http://uswest.ensembl.org/index.html) was used for differential ncRNAs screening. Furthermore, survival and survminer packages were used for survival curve analyses, and ggsurvplot package was used for visualization. Weighted Correlation Network Analysis was constructed to find downstream functional genes of RP9P and we used TCGA-Coadread and GSE121842 sequencing data to screen miRNAs associated with RP9P.

### Cell Culture

The human CRC cell lines, namely HCT116, SW620, HT29, and HCT8, and control NCM460 cells, were purchased from the Cell Bank of the Chinese Academy of Sciences. All cells were cultured in RPMI-1640 (PM150115, Procell, Wuhan, China) supplemented with 10% fetal bovine serum (085-060, WISENT, Nanjing, China) and the antibiotics penicillin and streptomycin (c125c5, NCM Biotech, Suzhou, China). Cells were cultured in a 37°C humidified incubator with 5% CO_2_.

### Cell Transfection

Specific short hairpin RNAs (shRNAs) against RP9P (shRP9P-1: GATCCGCTACAAAGAATGCCCTTTCTCTCGAGAGAAAGGGCATTCTTTGTAGCTTTTTG; shRP9P-2: GATCCGCACATGAGGATTTCATGTATCTCGAGATACATGAAATCCTCATGTGCTTTTTG) and *FOXQ1* (shFOXQ1: GATCCGCACATCATCCGGCACCATTTCTCGAGAAATGGTGCCGGATGATGTGCTTTTTG) were synthesized by Tsingke (Beijing, China). The miR-133a-3p mimic and inhibitor were purchased from General Biol (Anhui, China); their sequences were as follows: miR-133a-3p mimic: UUUGGUCCCCUUCAACCAGCUG; miR-133a-3p inhibitor: CAGCUGGUUGAAGGGGACCAAA. CRC cells were plated on 6-well plates to 60–70% confluence and transfected with Lipofectamine 2000 (11668019, Thermo Fisher Scientific, Waltham, MA, USA) according to the manufacturer’s instructions.

### Cell Counting Kit-8 Assay

Cell proliferation rates were detected by the CCK-8 assay (96992-100TESTS-F, Sigma-Aldrich, St. Louis, MO, USA). After digestion with 0.25% trypsin (c125c1, NCM Biotech), 5 × 10^3^ cells were plated in each well of a 96-well culture plate and incubated overnight at 37°C. At 0, 24, 48, and 72 h after transfection, 10 µL of the CCK-8 solution was added to each well and incubated for 2 h at 37°C. The optical density of each well was detected at 450 nm using a microplate reader (PerkinElmer, Waltham, MA, USA).

### Apoptosis Assays

Cell apoptosis was detected with the Annexin-V-FITC Apoptosis Detection Kit (KGA108-1, KeyGEN, China). Transfected cells were collected and resuspended in binding buffer. Next, cells were stained in sequence with 5 μL Annexin V-FITC and 5 μL propidium iodide for 15 min at room temperature in the dark. Lastly, the cells were subjected to flow cytometry analysis (ACEA Biosciences, San Diego, CA, USA). Collected data was analyzed with NovoExpress software.

### Nuclear and Cytoplasmic Fractions

A Cytoplasmic and Nuclear RNA Purification Kit (21000, Norgen, Belmont, CA, USA) was used to isolate and purify nuclear and cytoplasmic RNA fractions. Cells (3 × 10^6^) were lysed using lysis buffer on ice and then centrifuged at 14,000 × *g* for 10 min. The supernatant and pellet were separated to isolate the cytoplasmic and nuclear RNA fractions, respectively.

### Real-Time-PCR

Total RNA was isolated using TRIzol reagent (15596026, Thermo Fisher Scientific) and then reversed transcribed into cDNA using the RevertAid First Strand cDNA Synthesis Kit (qp006, GeneCopoeia, Guangzhou, China). Subsequently, RT-PCR was performed on a Fluorescence quantitative PCR instrument (qTower 3.2G, Analytik Jena, Thuringia, Germany). The RT-PCR protocol was as follows: 1) 95°C for 2 min, 2) 95°C for 15 s, 3) 60°C for 30 s, 4) repeat steps 1–3 for 40 cycles. All primers used for the RT‐PCR are listed in **Table 1**. The relative expression levels were counted by a 2^−ΔΔCt^ method.

**Table 1:** Primers used in this study.

Primer	Sequences (5’→3’)
GAPDH Forward Primer	ACAGCCTCAAGATCATCAGC
GAPDH Reverse Primer	GGTCATGAGTCCTTCCACGAT
RP9P Forward Primer	GGGTGGCAGGACTGGGAGAAT
RP9P Reverse Primer	CTCTCGCCTGTAATCCCAATGCT
FOXQ1 Forward Primer	ACCTTTCGCTCAACGACTGCT
FOXQ1 Reverse Primer	GGGGTTGAGCATCCAGTAGTTG
miR-133a-3p RT Primer	GTCGTATCCAGTGCGTGTCGTGGAGTCGGCAATTGCACTGGATACGACCAGCTG
miR-133a-3p Forward Primer	GCTTTGGTCCCCTTCAAC
miR-133a-3p Reverse Primer	CAGTGCGTGTCGTGGA
U6 Forward Primer	CTCGCTTCGGCAGCACA
U6 Reverse Primer	AACGCTTCACGAATTTGCGT

### Western Blotting Analyses

Tissue samples and cultured cells were lysed with a RIPA lysis buffer kit (P0013B, Beyotime, Haimen, China). Proteins were quantified using a bicinchoninic acid protein quantification kit (P0010S, Beyotime). Cell lysates were separated electrophoretically on 10% or 15% SDS-PAGE and transferred onto PVDF membranes (EMD Millipore, Billerica, MA, USA). These membranes were then incubated with rabbit monoclonal antibodies against BAX, BCL2, MCM2, PCNA, FOXQ1, and GAPDH (Proteintech, Rosemont, IL, USA) after being blocked with 5% skim milk for 2 h at room temperature. The membranes were then incubated with anti-rabbit IgG (H+L) (Cell Signaling Technology, Danvers, MA, USA) for 1 h at room temperature. Finally, signals were detected using an enhanced chemiluminescence kit (P10100, NCM Biotech). All results were analyzed using the ImageJ software (National Institutes of Health, Bethesda, MD, USA), and the densities of target bands were normalized to GAPDH.

### Immunohistochemical Staining

All specimens were fixed in 10% formaldehyde, paraffin embedded, and then sectioned at 4 µm. Paraffin slices were dewaxed and rehydrated, and antigen retrieval was performed using routine methods. Endogenous peroxidases were blocked with 3% H_2_O_2_. After the blocking step, sections were incubated with anti-MCM2, -PCNA, -BAX, and -BCL2 antibodies (Proteintech) overnight at 4°C and then with a secondary antibody (A0208, Beyotime) for 20 min. Subsequently, 3,3′-diaminobenzidine (P0202, Beyotime) and hematoxylin (BA4041, BASO, China) were used to visualize antigen–antibody complexes. A NEXcope microscope (Ningbo Yongxin Optics, Ningbo, China) was used to capture images.

### Dual-luciferase Reporter Assay

The binding sites between RP9P or *FOXQ1* and miR-133a-3p were analyzed using the Targetscan online software (http://www.targetscan.org/vert_72/). The 3′-untranslated region (UTR) or mutant gene fragments of RP9P or *FOXQ1* were inserted into the pGL3 promoter vector (Promega, Madison, WI) to construct wild-type and mutant reporter plasmids. For the luciferase reporter assay, CRC cells were co-transfected with the miR-133a-3p mimic, miR-133a-3p inhibitor, or miR nonsense control (NC) together with wild-type and mutant reporter plasmids for RP9P or *FOXQ1* using Lipofectamine 2000. Firefly and *Renilla* luciferase signals were measured 48 h after transfection using a Dual Luciferase Reporter Assay Kit (rg027, Beyotime), and the optical density of each well was detected using a microplate reader (PerkinElmer). Relative luciferase activity was normalized against *Renilla* luciferase activity.

### Tumorigenicity in Xenograft Nude Mice

Twelve male BALB/c nude mice (5 to 6 weeks old) were used in this experiment. After being divided randomly into RP9P and NC groups, the mice were subcutaneously injected with 5 × 10^6^ CRC cells to induce subcutaneous tumors. Tumor length (L) and width (W) were measured every 3 to 4 days once visible tumors appeared, and the tumor volume (V) was calculated according to the formula: V = L × W^2^/2. The mice were sacrificed after the experiment, and the tumor tissues were weighed and fixed in paraffin.

### Statistical Analyses

All data were analyzed with GraphPad Prism 8 software. Student’s *t*-test was conducted for comparisons between two groups. Comparisons among multiple groups were performed with a one- or two-way analysis of variance. Multi-weight comparisons were made using Dunnett’s test. Kaplan-Meier and log-rank tests were used for survival analyses. *p <*0.05 was considered statistically significant.

## Results

### RP9P is Highly Expressed in CRC and Correlates With Worse Prognosis

**Figure 1:**
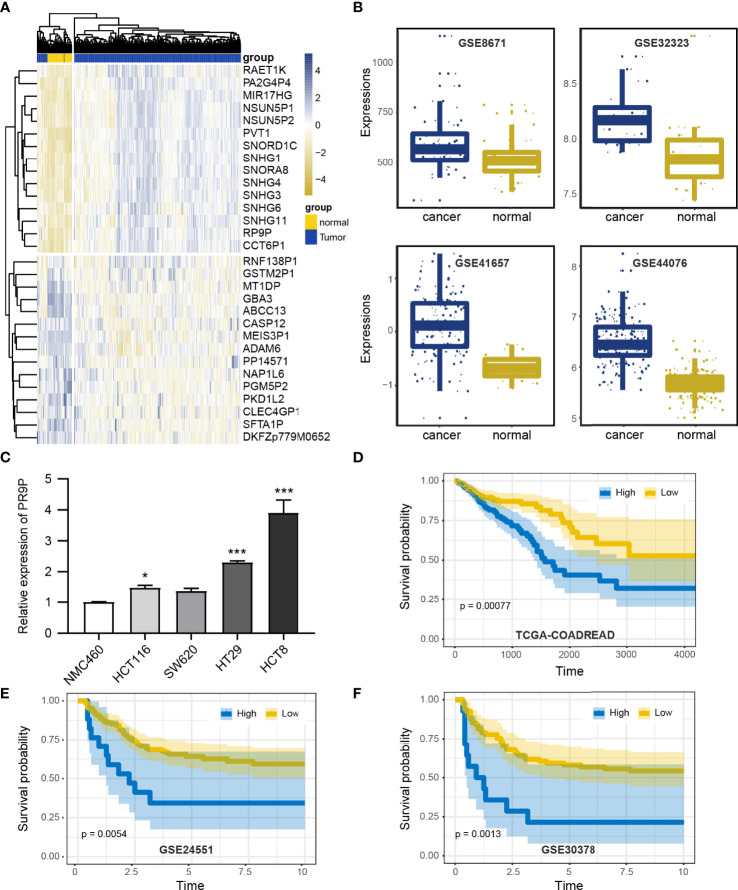
Expression of RP9P in colorectal cancer (CRC) based on The Cancer Genome Atlas (TCGA) database. **(A)** Heat map of different lncRNA expression levels in CRC detected using TCGA-COADREAD data. **(B)** RP9P level in CRC tissues compared with normal CRC tissues detected using GSE32323, GSE41657, GSE44076, and GSE8671 data. **(C)** RP9P level in CRC cell lines (HCT116, SW620, HT29, and HCT8) compared with the NMC460 cell line (*p < 0.05, ***p < 0.001, vs. NMC460) **(D–F)**. Correlation of the RP9P level with overall survival in CRC patients using the Kaplan-Meier analysis from TCGA-COADREAD **(D)**, GSE24551 **(E)**, and GSE30378 **(F)** data.

To investigate the effect of RP9P, we analyzed TCGA data (COADREAD) of 383 CRC and 51 non-cancer tissues. The RP9P mRNA level was markedly elevated in CRC compared to normal tissues (**Figure 1A**); this finding was verified in four other databases (**Figure 1B**). Cell experiments also showed that CRC cell lines had high RP9P expression compared to control NCM460 cells (**Figure 1C**). The survival analysis revealed that higher RP9P level was linked to poorer overall survival in CRC patients (*p* = 0.0092, **Figure 1D**); this finding was also verified by two other databases (**Figures 1E, F**). Therefore, RP9P overexpression might be a potential indicator for worse prognosis in CRC.

### RP9P Knockdown Inhibits Cell Proliferation and Induces Apoptosis in CRC Cells

**Figure 2:**
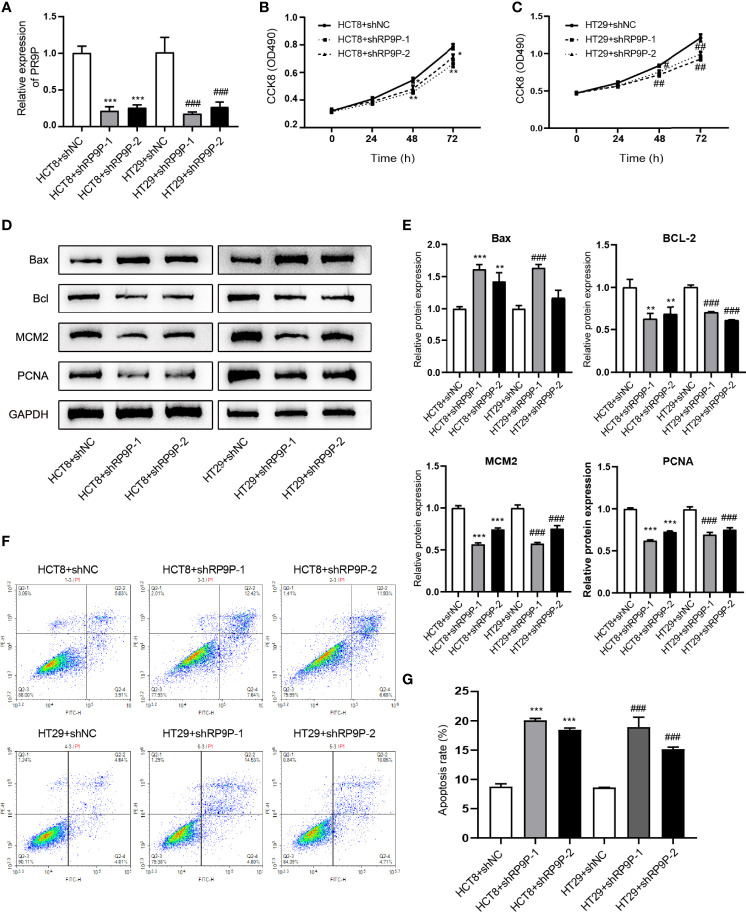
Effect of downregulated RP9P on colorectal cancer cell viability. **(A)** Expression of RP9P in HCT8 and HT29 cells with RP9P stably silenced was confirmed using real-time PCR. **(B, C)** HCT8 **(B)** and HT29 **(C)** cell proliferative ability with RP9P stably silenced was analyzed using the CCK-8 assay. **(D, E)** The levels of BAX, BCL2, MCM2, PCNA, and GAPDH were measured through western blotting **(D)**. Data are presented as means ± standard deviation **(E–G)** Percentages of apoptotic HCT8 and HT29 cells **(F)** were measured using flow cytometry. The histograms show the average numbers of apoptotic cells **(G)** (*p < 0.05, **p < 0.01, ***p < 0.001 vs. HCT8 + shNC; ^#^ *p* < 0.05, ^##^ *p* < 0.01, ^###^ *p* < 0.001 vs. HT29 + shNC).

Specific shRNAs were used to downregulate RP9P in HCT8 and HT29 cells. RT-PCR showed that these shRNAs decreased the RP9P mRNA level in HCT8 and HT29 cells (**Figure 2A**). To explore the function of RP9P, we measured the proliferation of HCT8 and HT29 cells following RP9P inhibition using the CCK-8 assay. RP9P knockdown significantly suppressed HCT8 and HT29 cell growth (**Figures 2B, C**). Western blotting showed that expression of the proliferation-related genes *MCM2* and *PCNA* decreased in lncRNA-shRNA cells (**Figures 2D, E**). Thus, RP9P has critical effects on the growth of CRC cells, and RP9P knockdown suppresses the proliferation of these cells.

We next determined the effects of RP9P on CRC cells using flow cytometry. As shown in **Figures 2F, G**, RP9P depletion promoted apoptosis in both HCT8 and HT29 cells. Western blotting showed that the apoptosis-related genes *BAX and BCL2*were increased and suppressed, respectively, in RP9P-knockdown CRC cells (**Figures 2D, E**). These results suggest that RP9P inhibits apoptosis of CRC cells.

### RP9P Inhibition Decreases Cell Growth in a Xenograft Model

**Figure 3:**
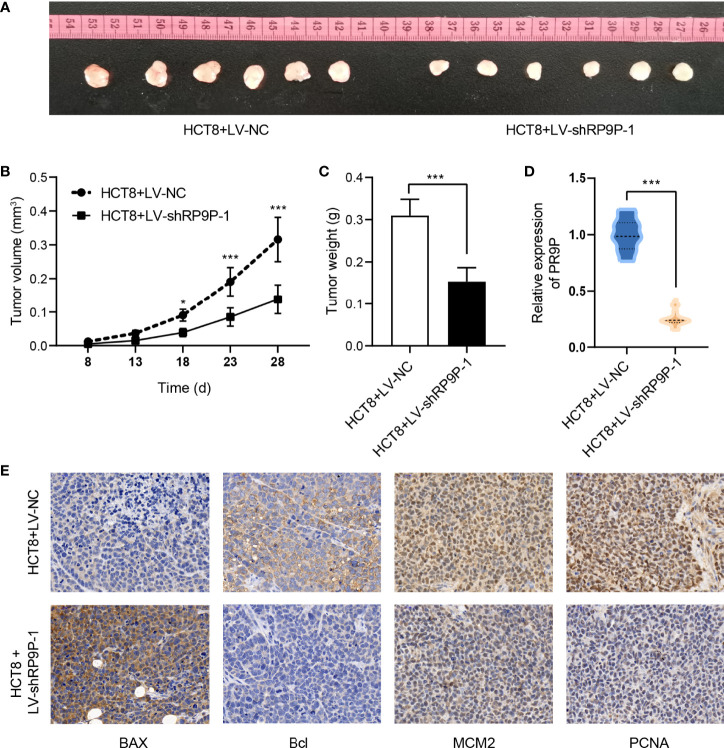
RP9P knockdown suppresses colorectal cancer cell proliferation in a xenograft model. **(A)** Representative images of transplanted tumors in nude mice. **(B, C)** Tumor volume **(B)** and tumor weight **(C)**. **(D)** RP9P level in transplanted tumors. **(E)** Levels of proliferation- and apoptosis-related genes were examined by immunohistochemistry (*p < 0.05, ***p < 0.001).

To confirm the role of RP9P in CRC *in vivo*, we injected HCT8 cells with stable silenced RP9P, or control cells, subcutaneously into BALB/c nude mice to generate a xenograft model. As shown in **Figures 3A–E**, tumors with RP9P knockdown had a smaller size and lower weight compared to controls. Furthermore, downregulation of RP9P expression in RP9P-knockdown cell tumors was confirmed with the RT-PCR assay. The results of immunohistochemistry indicated that the expression of the apoptosis-related genes *BAX* and *BCL2* increased and decreased, respectively, whereas that of the proliferation-related genes *MCM2* and *PCNA* decreased in RP9P-knockdown cell tumors. These findings suggest that knockdown of RP9P inhibits CRC cell growth *in vivo*.

### miR-133a-3p Is a Downstream Target of RP9P

**Figure 4:**
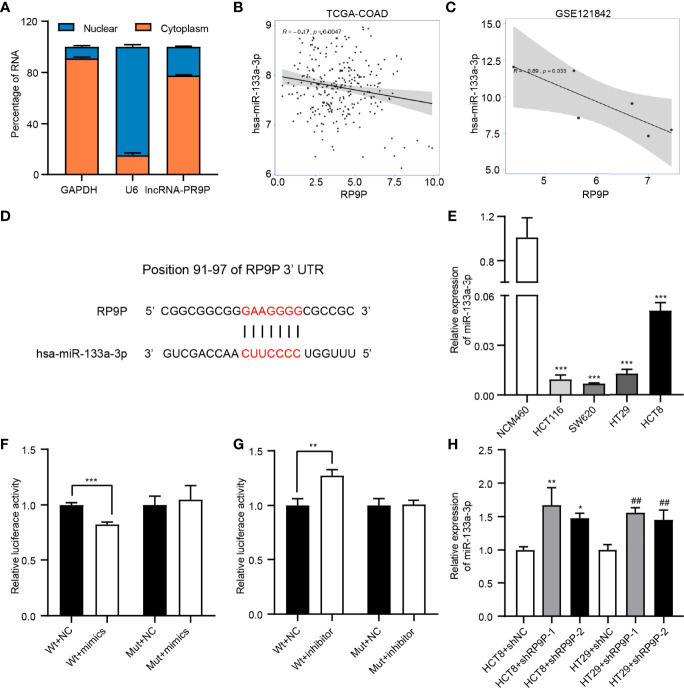
Identification of miR-133a-3p as a direct target gene of RP9P. **(A)** Subcellular localization of RP9P in colorectal cancer (CRC) cells. **(B, C)** The relationship between RP9P and miR-133a-3p was determined using TCGA-COADREAD **(B)** and GSE121842 **(C)** data. **(D)** miR-133a-3p level in CRC cells identified using real-time (RT)-PCR (***p < 0.001 vs. NCM460 cells). **(E)** Predicted binding sites of RP9P and miR-133a-3p and mutated sites in the 3′-untranslated region (UTR) of RP9P. **(F, G)** Luciferase activity in nonsense control (NC)-, miR-133a-3p mimic- **(F)**, and miR-133a-3p inhibitor **(G)**‐treated CRC cells with wild-type or mutant RP9P 3′-UTR gene fragments (**p < 0.01, ***p < 0.001). **(H)** The miR-133a-3p level in CRC cells with RP9P silenced was examined using RT-PCR (*p < 0.05, **p < 0.01 vs. HCT8 + shNC; ^##^ *p* < 0.01 vs. HT29 + shNC).

We detected the subcellular localization of RP9P in CRC cells to investigate its mechanisms. The results of the subcellular fractionation assay revealed that more than 70% of RP9P was distributed in the cytoplasm (**Figure 4A**). As lncRNAs are associated with miRNAs, we used TCGA-COADREAD and GSE121842 data to screen miRNAs related to RP9P. The results revealed that RP9P might directly target miR-133a-3p (**Figures 4B, C**). Potential binding sites were predicted using starBase software (**Figure 4D**), and the low level of miR-133a-3p was confirmed in CRC cell lines (**Figure 4E**). The dual-luciferase reporter assay showed that the miR-133a-3p mimic remarkably decreased (**Figure 4F**) while the inhibitor increased (**Figure 4G**) the relative luciferase intensity of the reporter containing wild-type RP9P in CRC cells; however, no significant changes were found in the mutant group. Furthermore, knockdown of RP9P upregulated miR-133a-3p expression in CRC cells (**Figure 4H**). These findings show that RP9P targets miR-133a-3p.

### The miR-133a-3p Inhibitor Restored the Reduced CRC Cell Viability Caused by shRP9P

**Figure 5:**
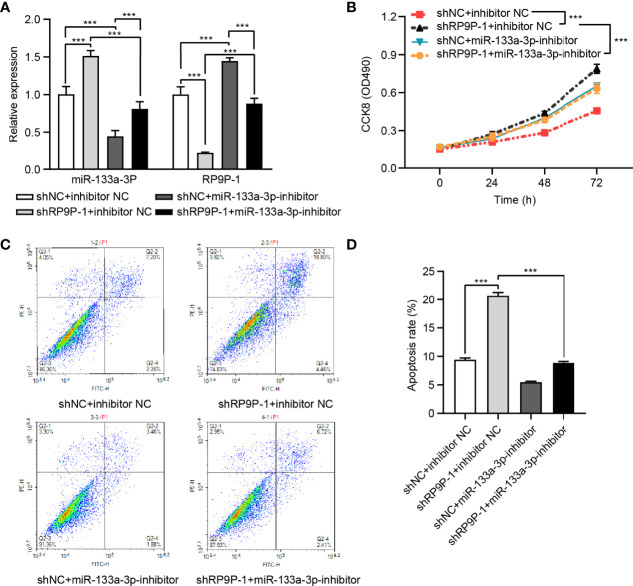
RP9P affects colorectal cancer (CRC) cell viability *via* miR-133a-3p. **(A)** RP9P and miR-133a-3p levels in shRP9P-, miR-133a-3p inhibitor-, or nonsense control (NC)- transfected CRC cells. **(B)** Cell proliferation was measured using the CCK-8 assay. **(C, D)** Apoptosis after cell transfection was analyzed using flow cytometry **(C)**. The histograms show the average numbers of apoptotic cells **(D)** (***p < 0.001).

To confirm the influence of miR-133a-3p on the function of RP9P, shRP9P and the miR-133a-3p inhibitor or NC were co-transfected into HCT8 cells. RP9P and miR-133a-3p levels in shRP9P-, miR-133a-3p inhibitor-, or nonsense control (NC)- transfected CRC cells were confirmed using RT-PCR assay (**Figure 5A**). CCK-8 and flow cytometry assay results showed that the miR-133a-3p inhibitor alleviated the anti-proliferation effect of RP9P knockdown (**Figure 5B**) while restoring its pro-apoptotic effect (**Figures 5C, D**). Altogether, the above results indicate that RP9P promotes the viability of CRC cells by targeting miR-133a-3p *via* a sponge mechanism.

### miR-133a-3p Directly Binds FOXQ1 and Affects Its Function

**Figure 6:**
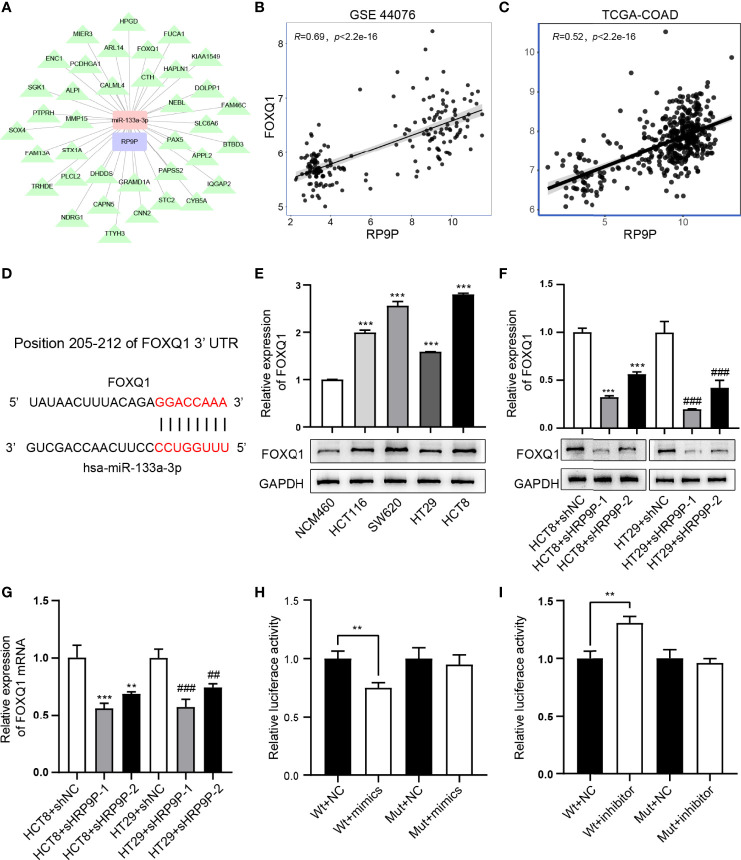
miR-133a-3p directly binds to FOXQ1. **(A)** Target genes of miR-133A-3p with RP9P as the center were constructed using cytoscape. **(B, C)** The relationship between FOXQ1 and miR-133a-3p was analyzed using GSE44076 **(B)** and TCGA-COADREAD **(C)** data. **(D)** Predicted binding sites of miR-133a-3p and FOXQ1, and mutated sites in the 3′-untranslated region (3′-UTR) of *FOXQ1*. **(E)** FOXQ1 level in colorectal cancer (CRC) cell lines detected through western blotting (***p < 0.001 vs. NCM460 cells). F,G. Protein and mRNA expression of FOXQ1 in CRC cells with RP9P knockdown was detected using western blotting **(F)** and real-time PCR **(G)**, respectively (**p < 0.01, ***p < 0.001 vs. HCT8 + shNC; ^##^ *p* < 0.01, ^###^ *p* < 0.001 vs. HT29 + shNC). H,I. Luciferase activity in nonsense control (NC)-, miR-133a-3p mimic- **(H)**, and miR-133a-3p inhibitor **(I)**-treated CRC cells with wild-type or mutant *FOXQ1* 3′-UTR gene fragments (**p < 0.01).

Previous results have demonstrated that RP9P can function as a ceRNA by binding miR-133a-3p. Thus, we investigated the target of miR-133a-3p (**Figure 6A**). FOXQ1, which is positively correlated with RP9P (**Figures 6B, C**), has a potential miR-133a-3p-binding region (**Figure 6D**). The RT-PCR assay indicated that the *FOXQ1* mRNA level was higher in CRC cells than that in NCM460 cells (**Figure 6E**). The dual-luciferase reporter assay revealed that the miR-133a-3p mimic significantly reduced and the inhibitor increased the luciferase intensity of the reporter containing wild-type *FOXQ1* in CRC cells, while no significant change was found in the mutant group (**Figures 6H, I**).

**Figure 7:**
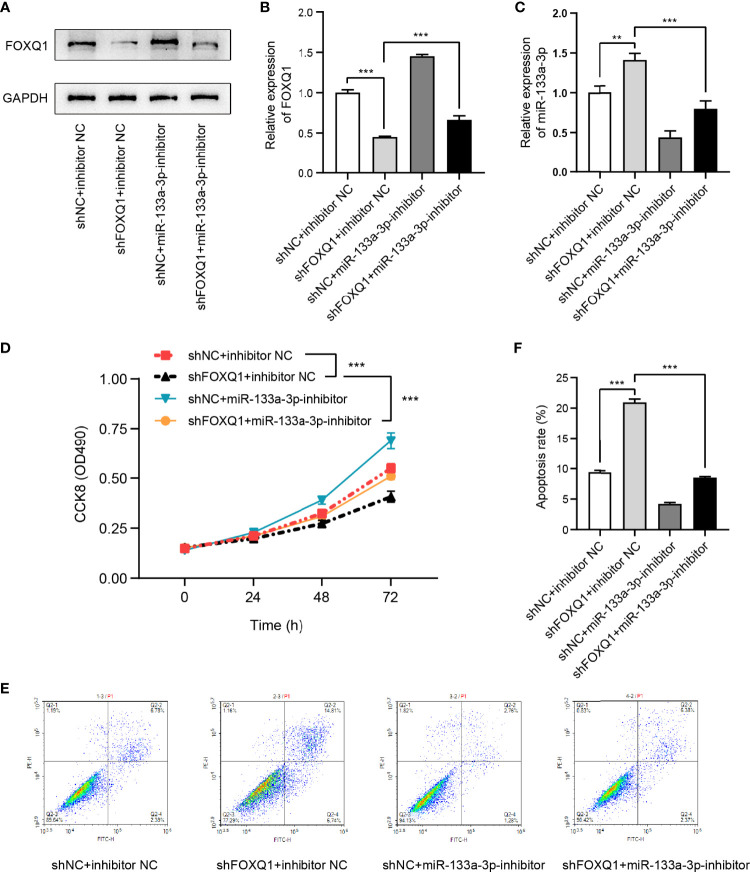
FOXQ1 affects colorectal cancer (CRC) cell viability *via* miR-133a-3p. **(A–C)**. Expression of FOXQ1 **(A, B)** and miR-133a-3p **(C)** in miR-133a-3p inhibitor-, shFOXQ1-, or nonsense control (NC)-transfected CRC cells. **(D)** Cell proliferation was measured using the CCK-8 assay. **(E, F)**. Apoptosis following cell transfection was analyzed through flow cytometry **(E)**. Histograms showing the average numbers of apoptotic cells **(F)** (**p < 0.01, ***p < 0.001).

To probe the influence of miR-133a-3p and FOXQ1 on CRC cell viability, the CCK-8 and apoptosis assays were performed. The expressions of FOXQ1 and miR-133a-3p in miR-133a-3p inhibitor-, shFOXQ1-, or nonsense control (NC)-transfected CRC cells were shown in **Figures 7A–C**. The results suggested that inhibiting miR-133a-3p blocked the negative effect of shFOXQ1 on HCT8 cell proliferation (**Figure 7D**), while it reversed the pro-apoptotic effect on CRC cells (**Figures 7E, F**). Furthermore, knockdown of RP9P suppressed the *FOXQ1* level in CRC cells (**Figures 6E–G**), consistent with previous findings. In summary, these results indicate that silencing miR-133a-3p enhances CRC cell viability by diminishing binding to FOXQ1.

## Discussion

Numerous studies have demonstrated that lncRNAs are closely related to CRC pathogenesis and development (12). For instance, the lncRNA SATB2-AS1 is low expressed in CRC and can regulate the function of immune cells by targeting SATB2 (13). The lncRNA LINRIS interacts with IGF2BP2 and affects glycolysis in CRC cells (14), whereas GAS5 inhibits progression of CRC by regulating phosphorylation of YAP (15). Here, we evaluated the function of RP9P in CRC. RP9P is a pseudogene-derived lncRNA, located at chromosome 7:32,916,815-32,943,176, that has never been reported previously in cancer. We found that the RP9P level was higher in CRC tissues, and the high level of RP9P was associated with worse prognosis. Furthermore, knockdown of RP9P *in vitro* repressed CRC cell growth. We also confirmed that downregulating the RP9P level suppressed tumor growth in xenograft nude mice. In summary, this study reveals that RP9P has pro-tumorigenic effects in CRC.

lncRNAs play different functional roles according to their locations (3). Growing evidence indicates that cytoplasmic lncRNAs can serve as miRNA sponges, modulating the mRNA level by binding to miRNAs (6). Here, the results of subcellular fractionation assay suggest that RP9P may act as a ceRNA in the cytoplasm of CRC cells. To verify this supposition, we attempted to identify miRNAs that could bind to RP9P. The bioinformatics analysis predicted that miR-133a-3p had an interaction effect with RP9P. The functions of miR-133a-3p in cancers have been widely reported. miR-133a-3p is decreased in CRC and is considered to exert a tumor suppressor effect by targeting SENP1 (16). miR-133a-3p can also inhibit the proliferation and autophagy in gastric cancer (17). In addition, miR-133a-3p decreases viability, migration, and invasion in gallbladder carcinoma (5).

The expression of RP9P and miR-133a-3p were inverse in CRC cell lines, in a similar fashion to the bioinformatics analysis. We confirmed a direct interaction between miR-133a-3p and RP9P. Additionally, the miR-133a-3p level was decreased by RP9P in CRC cells. Furthermore, downregulating miR-133a-3p expression rescued the tumor-suppressive effect caused by RP9P knockdown alone. These results indicate that RP9P promotes CRC progression *via* sponging miR-133a-3p.

Our further exploration with a bioinformatics analysis indicated that FOXQ1 is a downstream molecular target of miR-133a-3p. FOXQ1, a member of the large forkhead transcription factor family, is expressed at a high level in CRC and exerts as an oncogene. Previous study has shown that miR-133 exhibits tumor inhibition by directly binding FOXQ1 in lung cancer (18). This study confirmed that FOXQ1 can directly interact with miR-133a-3p in CRC. Functional experiments showed that miR-133a-3p knockdown rescued the anti-proliferation and pro-apoptotic effects induced by inhibiting FOXQ1 alone. Consistent with these results, RP9P knockdown also decreased the FOXQ1 level in CRC. Therefore, miR-133a-3p performs its functions dependent on FOXQ1 in CRC. However, the effects of RP9P on the function of FOXQ1 is unknow, which needs further experimental researches.

Taken together, our research reveals that RP9P is expressed at a low level in CRC and is involved in cell proliferation and apoptosis. RP9P promotes FOXQ1 expression by directly targeting miR-133a-3p *via* a sponge mechanism. Finally, this study indicates that RP9P is a novel oncogenic lncRNA in CRC that can be used as a therapeutic target.

## Data Availability Statement

The datasets presented in this study can be found in online repositories. The names of the repository/repositories and accession number(s) can be found in the article/supplementary material.

## Ethics Statement

The animal study was reviewed and approved by the Experimental Animal Ethics Committee of Jiangsu Province Hospital of Chinese Medicine.

## Author Contributions

JQ and ZJ conceived and designed the study. ZJ and BXL wrote the manuscript. ZJ and BFL performed the *in vitro* experiment. RY and CW performed the *in vivo* experiment. XZ revised the manuscript. WX performed the statistical analysis. All authors have read and approved the contents of the final manuscript.

## Funding

This research was supported by funds from National Natural Science Foundation of China (81904110, 82104950), Science and Technology Planning Project of Jiangsu Province, China [Grant number BK20191086], Developing Program for High-level Academic Talent in Jiangsu Hospital of TCM (Grant No. y2021rc46), Project of National Clinical Research Base of Traditional Chinese Medicine in Jiangsu Province, China (JD2019SZXYB01) and Medical Scientific Research Project of Jiangsu Health Commission (H2019094).

## Conflict of Interest

The authors declare that the research was conducted in the absence of any commercial or financial relationships that could be construed as a potential conflict of interest.

## Publisher’s Note

All claims expressed in this article are solely those of the authors and do not necessarily represent those of their affiliated organizations, or those of the publisher, the editors and the reviewers. Any product that may be evaluated in this article, or claim that may be made by its manufacturer, is not guaranteed or endorsed by the publisher.
