# Supplementary figures and images for: The Novel lncRNA RP9P Promotes Colorectal Cancer Progression by Modulating miR-133a-3p/FOXQ1 Axis
Source: Front Oncol. 2022 May 5;12:843064. doi: 10.3389/fonc.2022.843064 (PMC9117648; doi:10.3389/fonc.2022.843064)

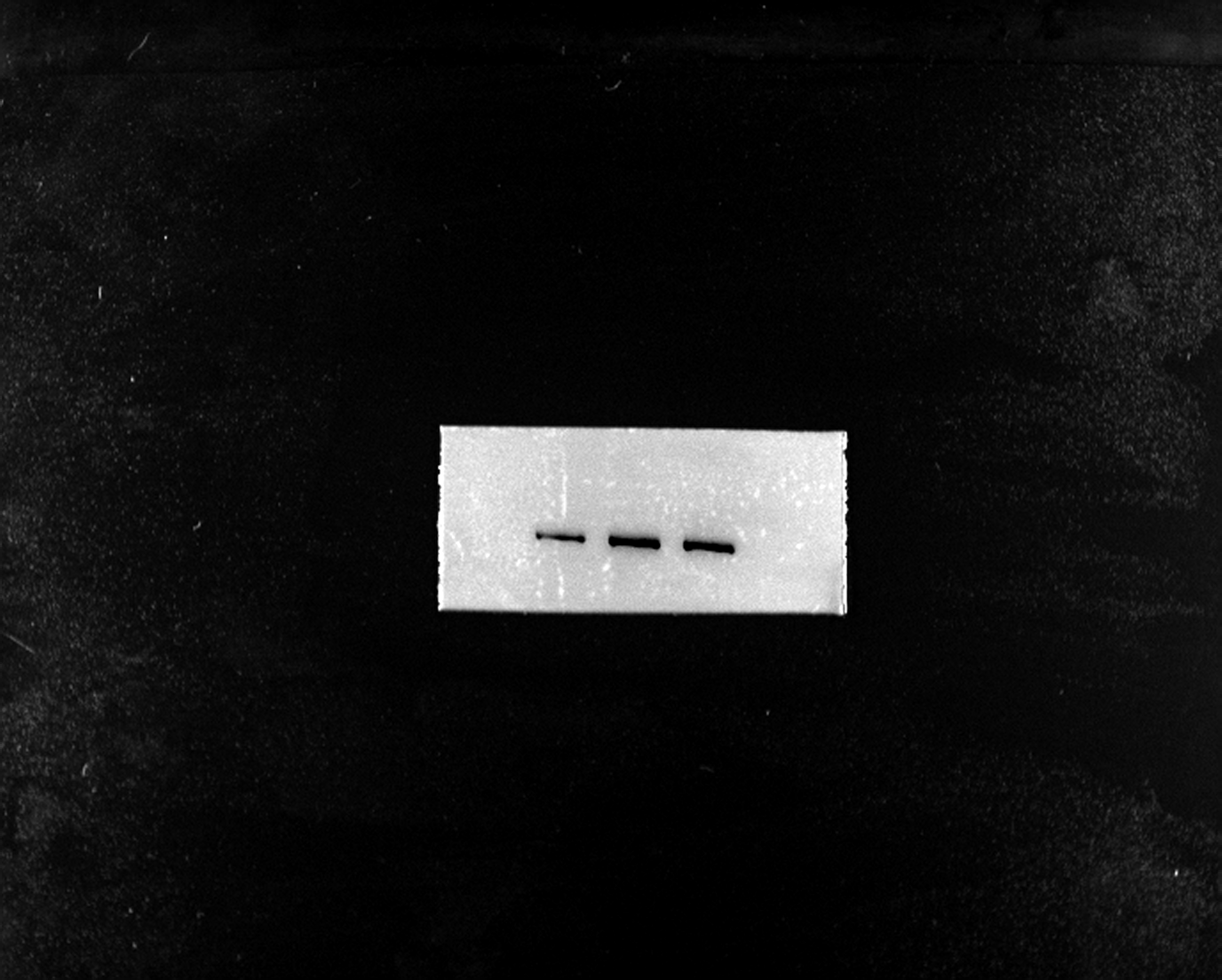

Supplement: Supplementary file 1 [file DataSheet_1.zip › WBs/2/1 Fig-2D-Baxú¿ó+HCT8+shNCú+ó+HCT8+shRP9P-1ú+ó¦HCT8+shRP9P-2ú¬.tif]

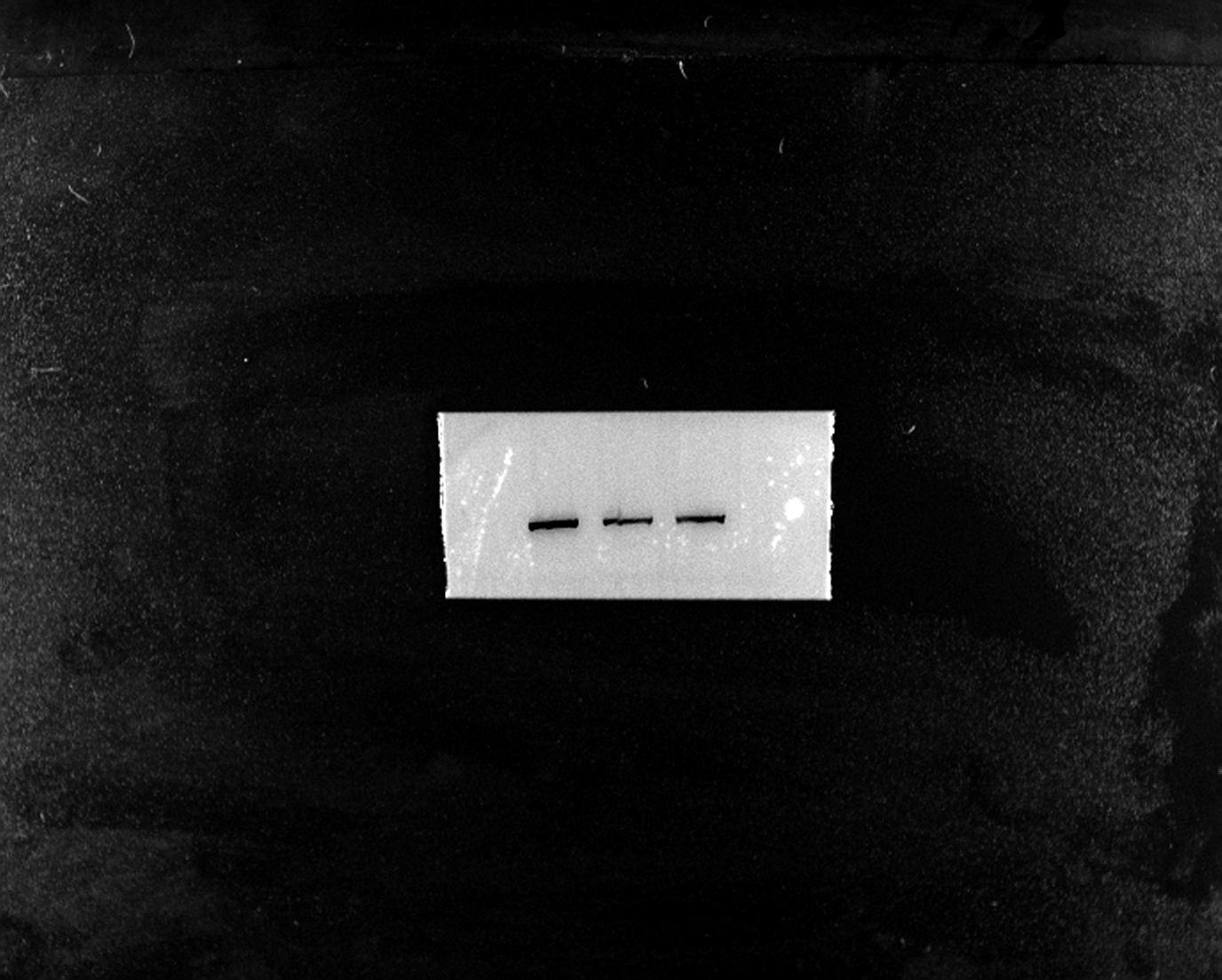

Supplement: Supplementary file 1 [file DataSheet_1.zip › WBs/2/2 Fig-2D-Bcl2ú¿ó+HCT8+shNCú+ó+HCT8+shRP9P-1ú+ó¦HCT8+shRP9P-2ú¬.tif]

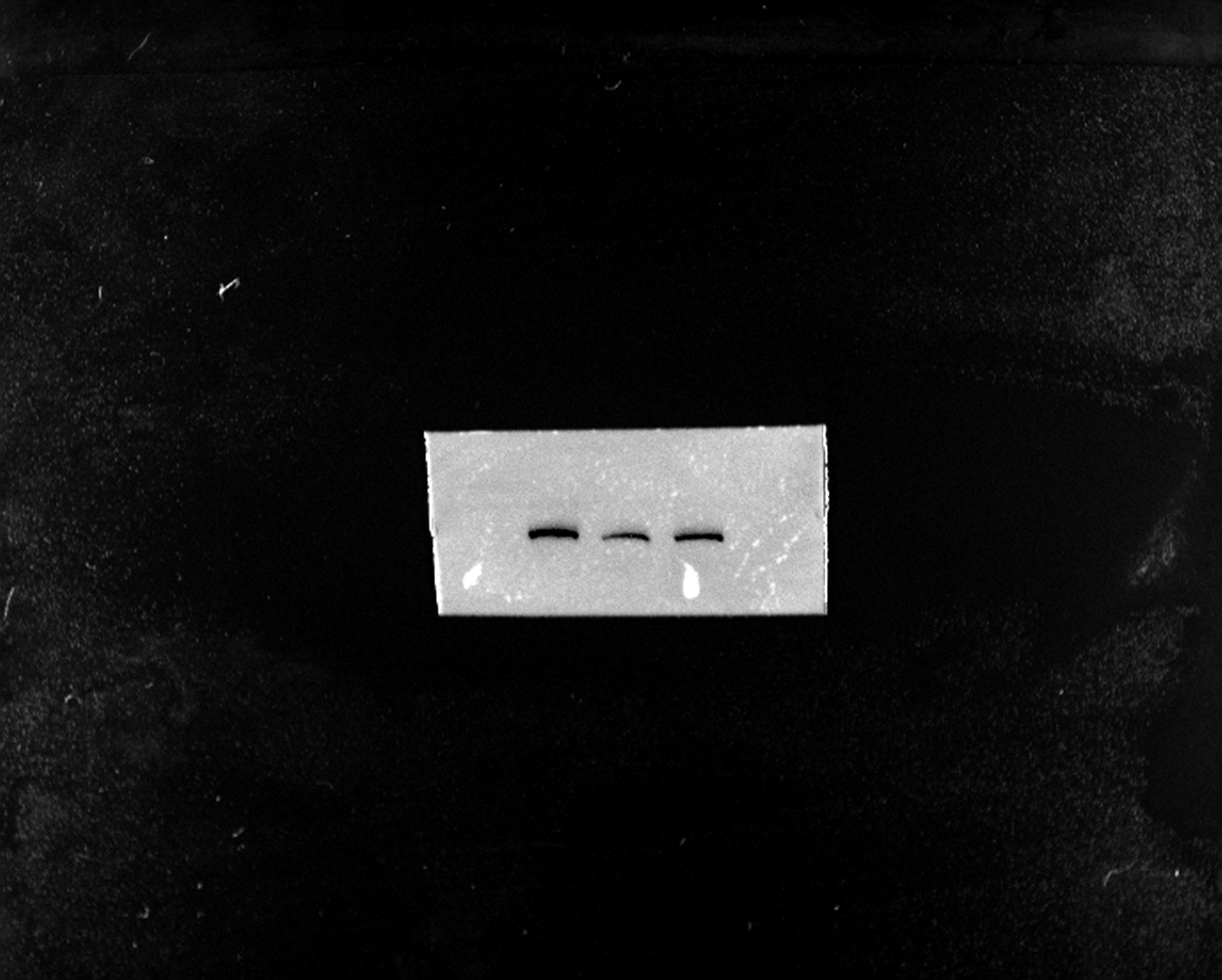

Supplement: Supplementary file 1 [file DataSheet_1.zip › WBs/2/3 Fig-2D-MCM2ú¿ó+HCT8+shNCú+ó+HCT8+shRP9P-1ú+ó¦HCT8+shRP9P-2ú¬.tif]

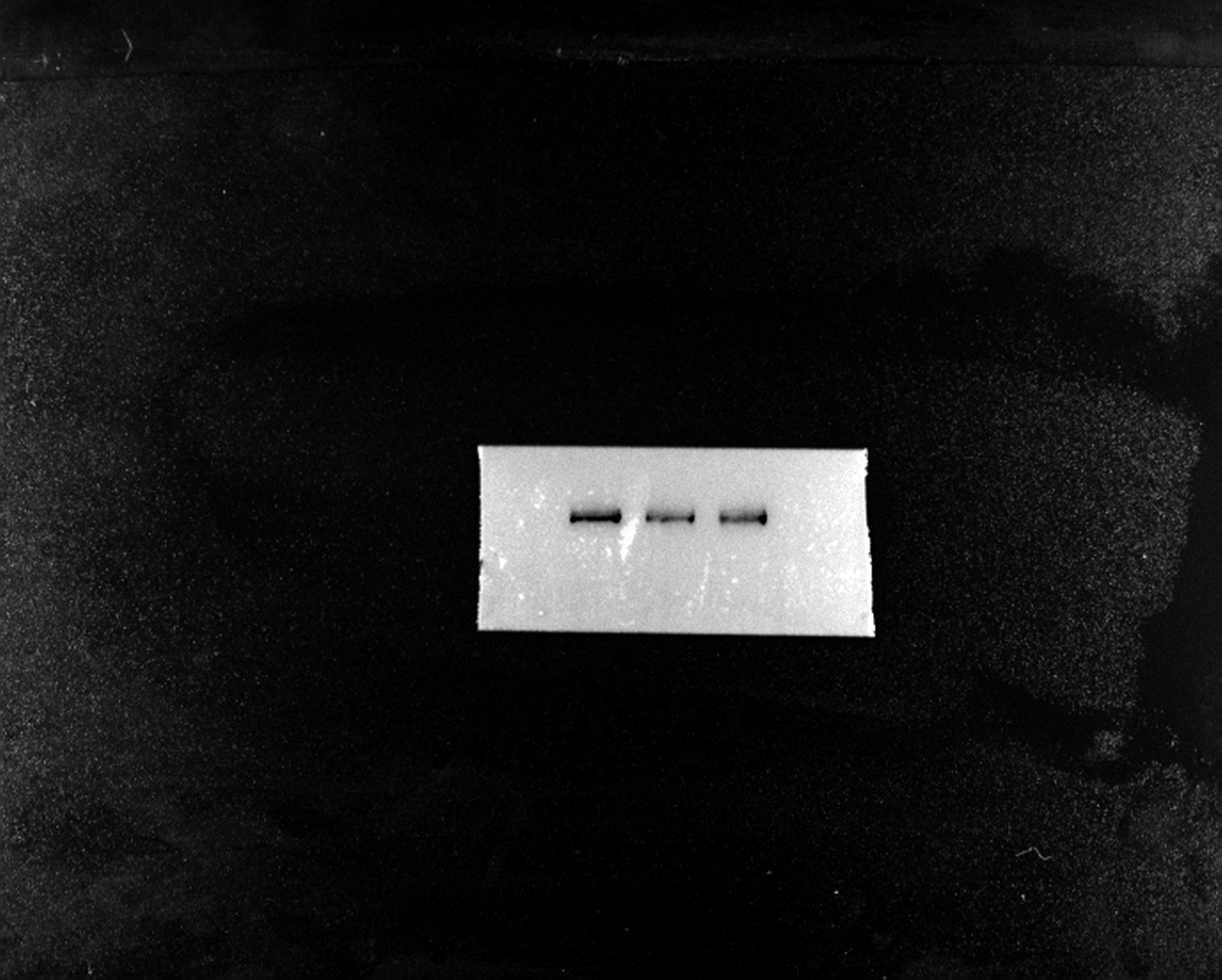

Supplement: Supplementary file 1 [file DataSheet_1.zip › WBs/2/4 Fig-2D-PCNAú¿ó+HCT8+shNCú+ó+HCT8+shRP9P-1ú+ó¦HCT8+shRP9P-2ú¬.tif]

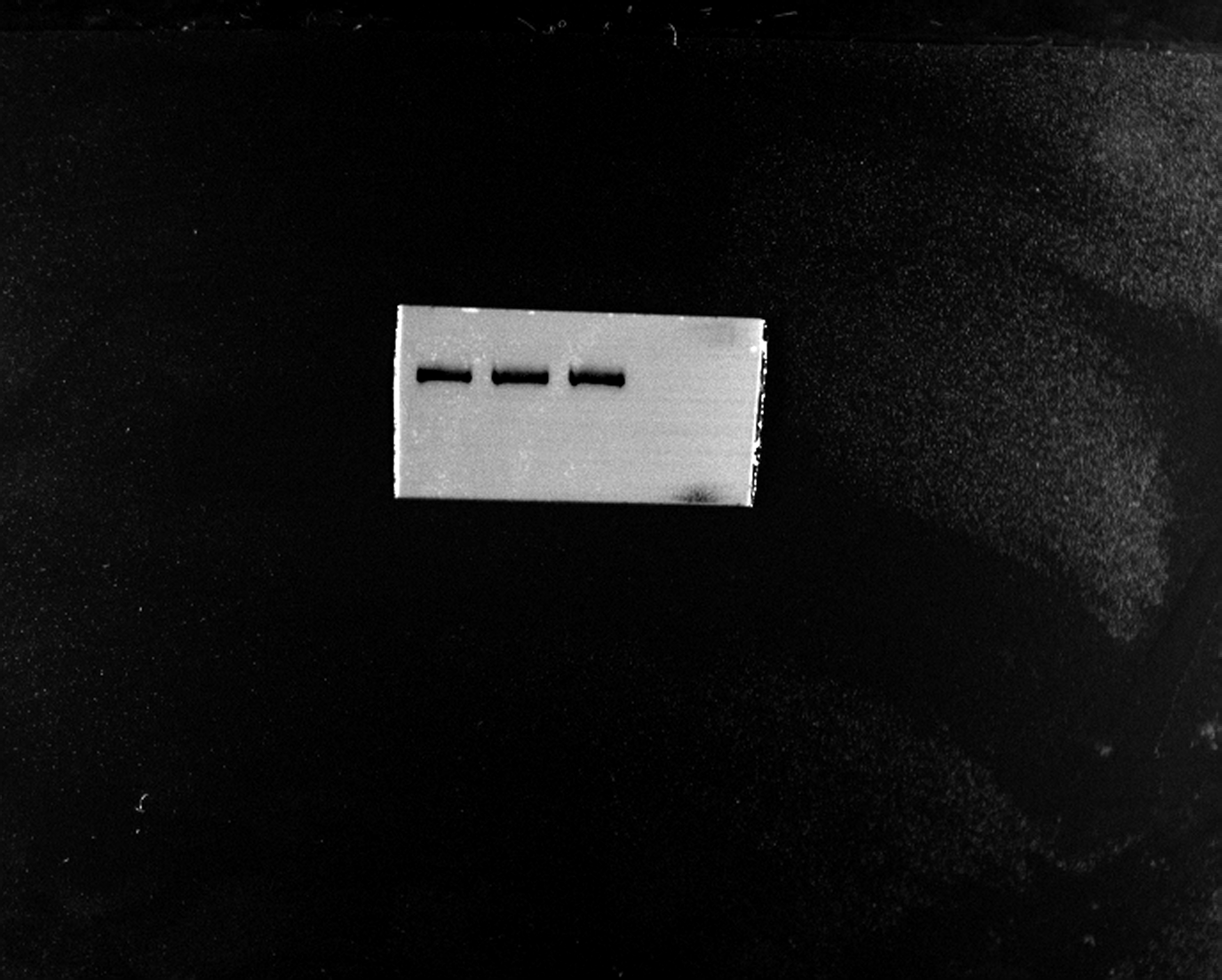

Supplement: Supplementary file 1 [file DataSheet_1.zip › WBs/2/5 Fig-2D-GAPDHú¿ó+HCT8+shNCú+ó+HCT8+shRP9P-1ú+ó¦HCT8+shRP9P-2ú¬.tif]

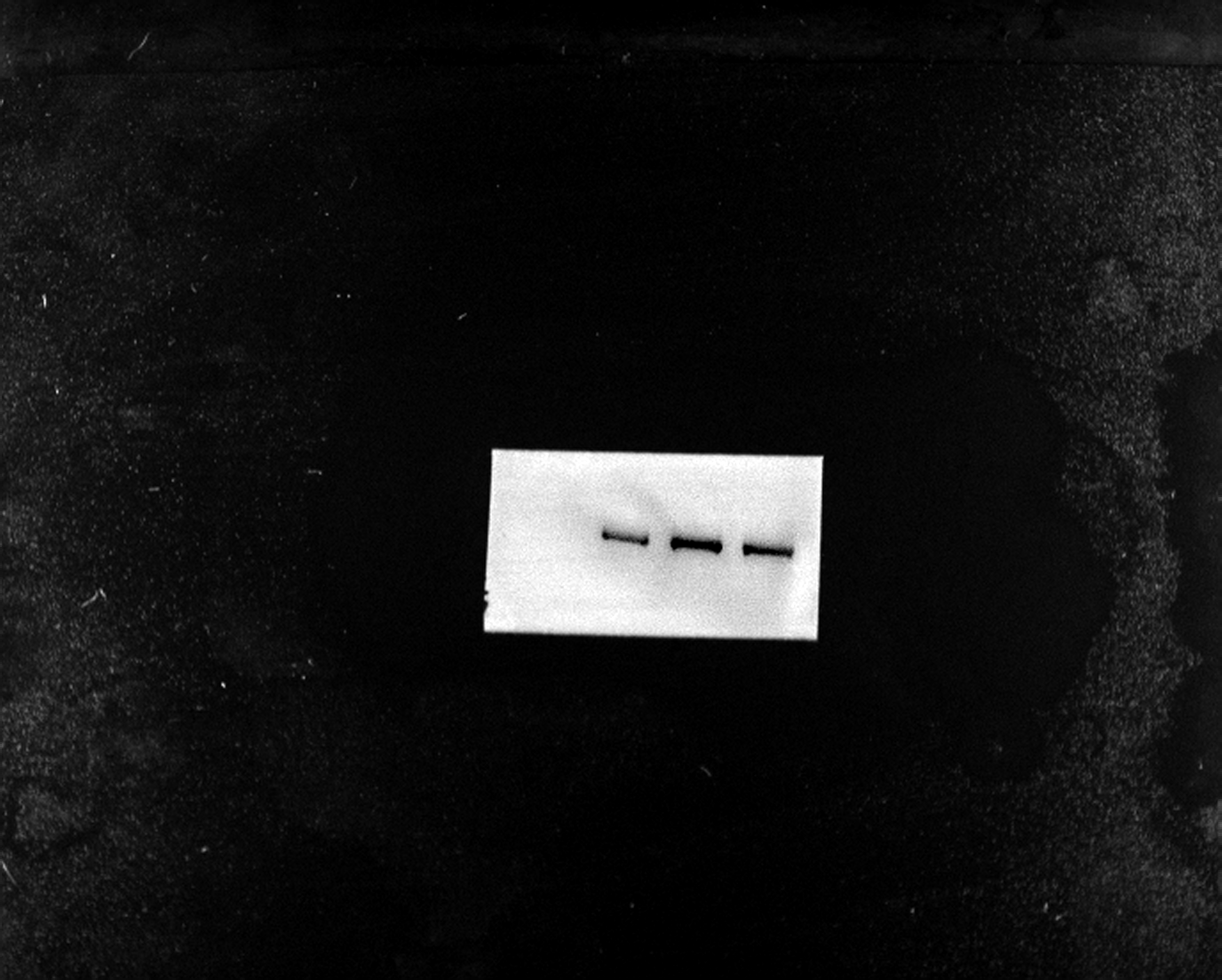

Supplement: Supplementary file 1 [file DataSheet_1.zip › WBs/2/6 Fig-2D-Baxú¿ó_HT29+shNCú+ó¦HT29+shRP9P-1ú+ó¦HT29+shRP9P-2ú¬.tif]

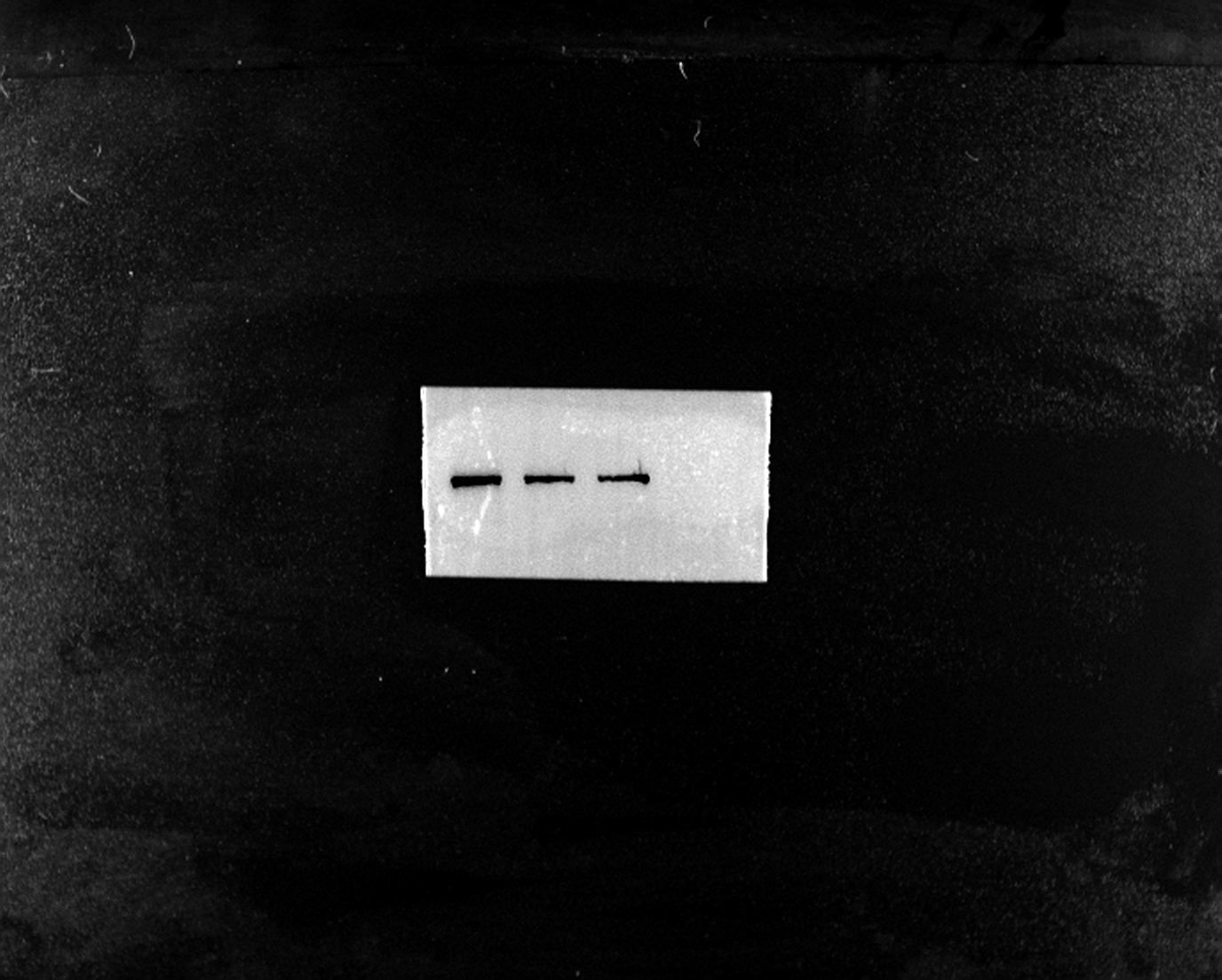

Supplement: Supplementary file 1 [file DataSheet_1.zip › WBs/2/7 Fig-2D-Bcl2ú¿ó_HT29+shNCú+ó¦HT29+shRP9P-1ú+ó¦HT29+shRP9P-2ú¬.tif]

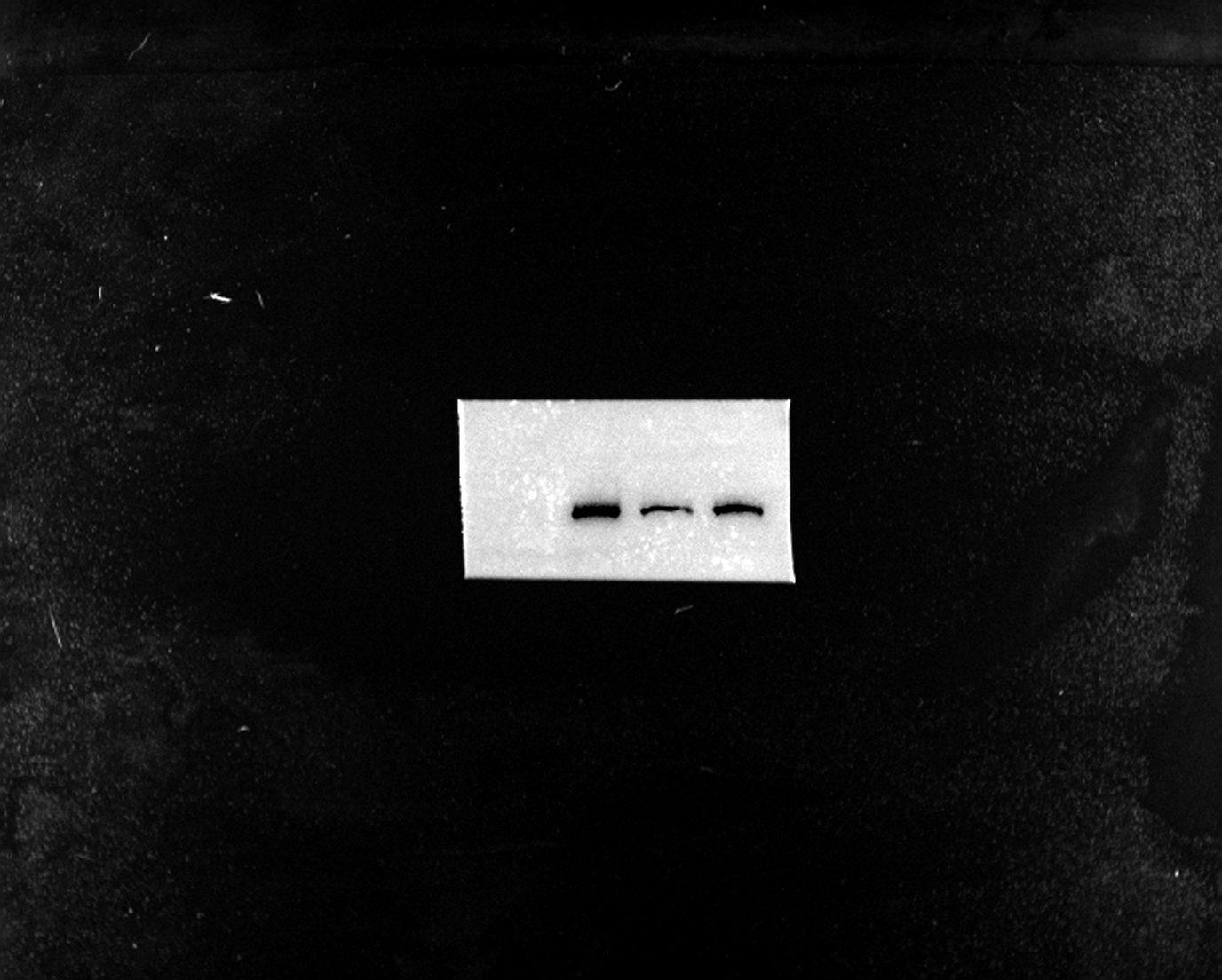

Supplement: Supplementary file 1 [file DataSheet_1.zip › WBs/2/8 Fig-2D-MCM2ú¿ó_HT29+shNCú+ó¦HT29+shRP9P-1ú+ó¦HT29+shRP9P-2ú¬.tif]

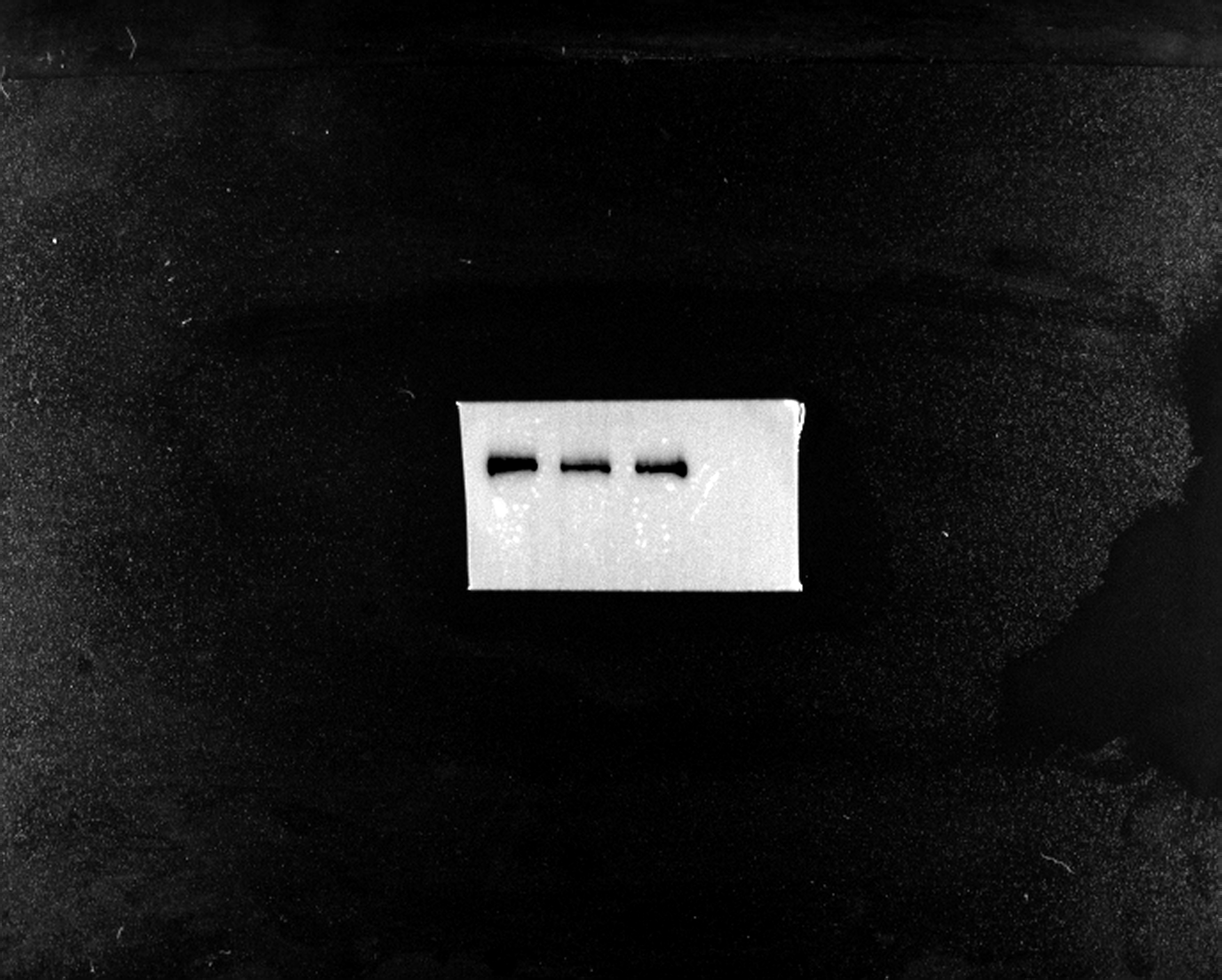

Supplement: Supplementary file 1 [file DataSheet_1.zip › WBs/2/9 Fig-2D-PCNAú¿ó_HT29+shNCú+ó¦HT29+shRP9P-1ú+ó¦HT29+shRP9P-2ú¬.tif]

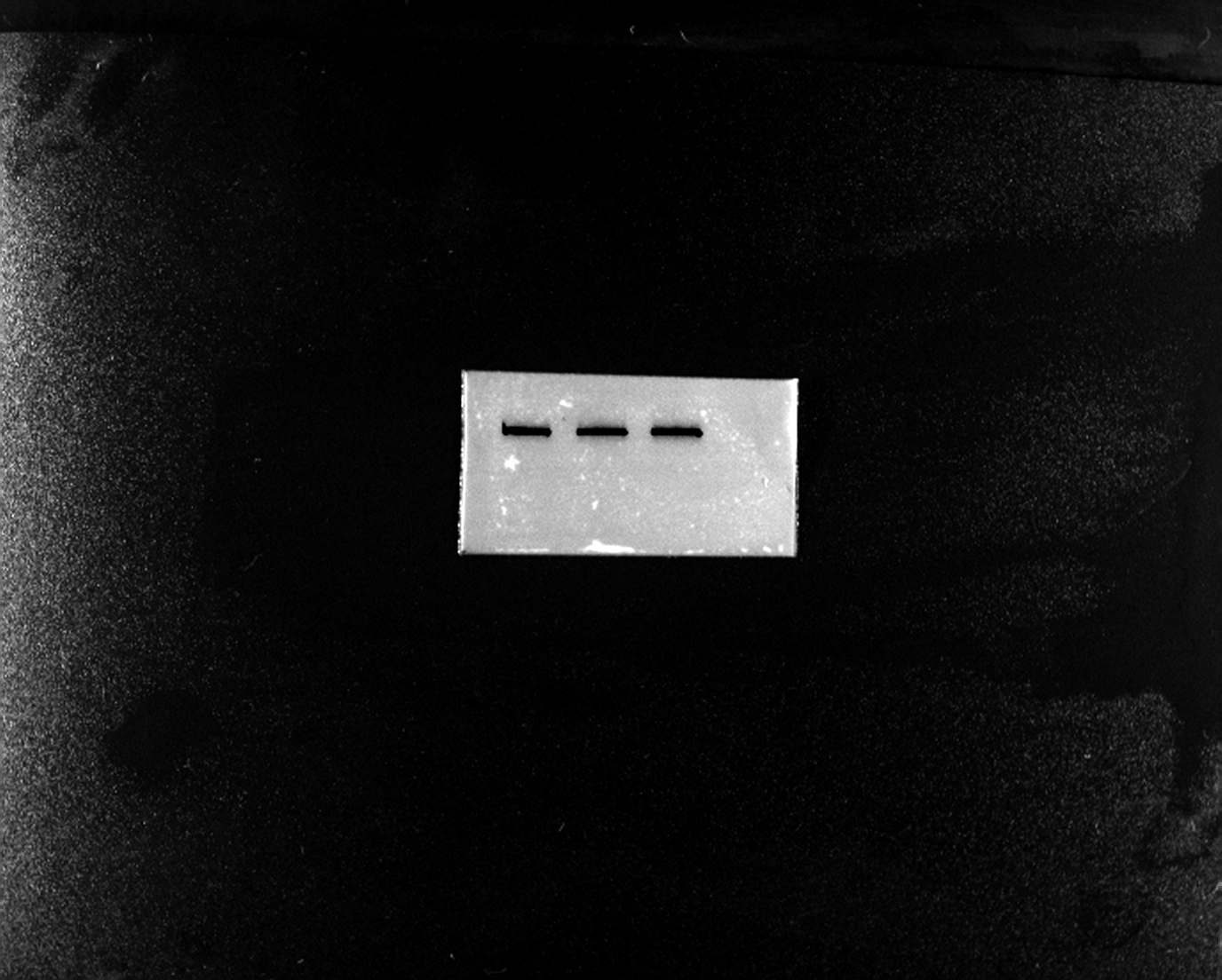

Supplement: Supplementary file 1 [file DataSheet_1.zip › WBs/2/Fig-2D-GAPDHú¿ó_HT29+shNCú+ó¦HT29+shRP9P-1ú+ó¦HT29+shRP9P-2ú¬.tif]

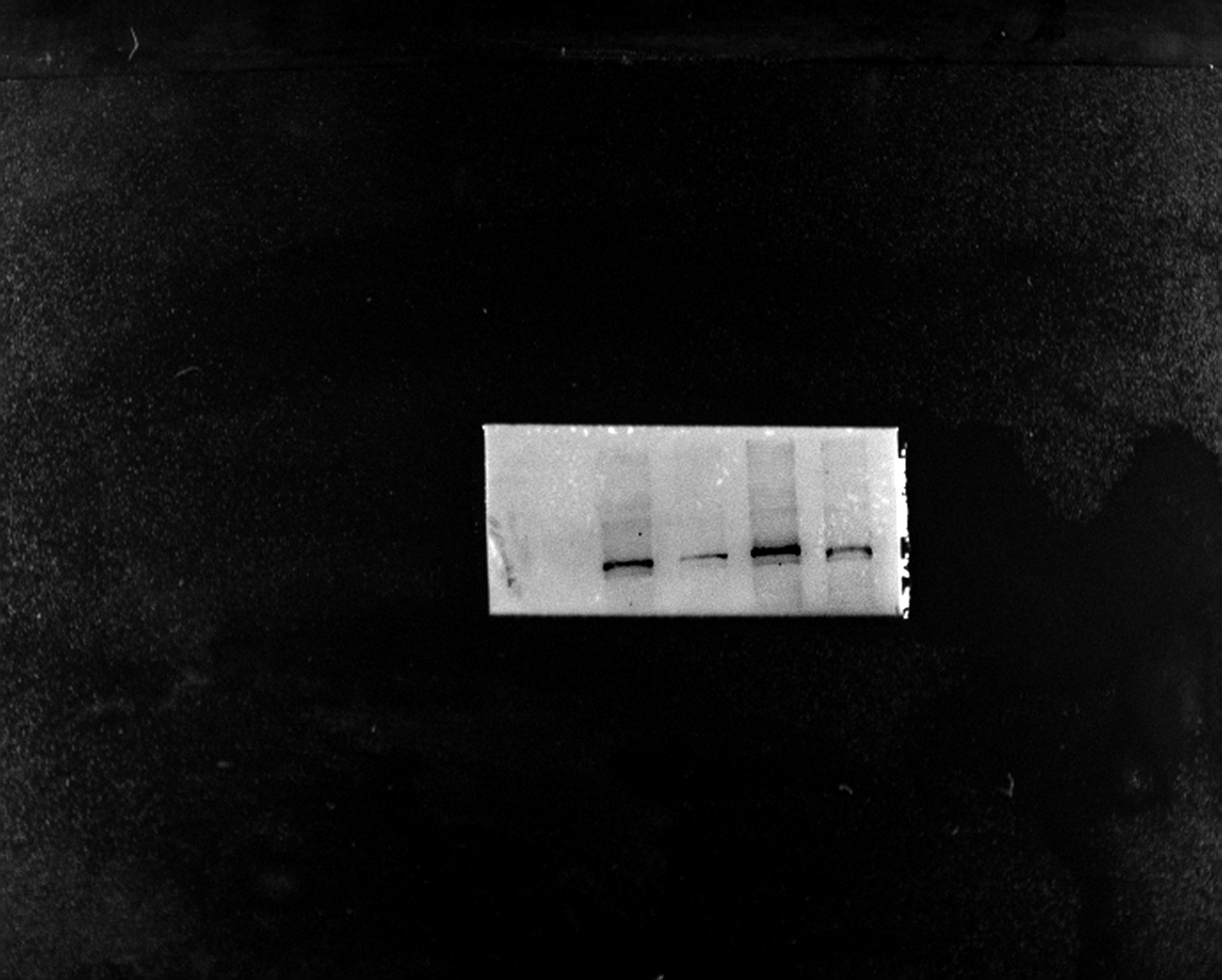

Supplement: Supplementary file 1 [file DataSheet_1.zip › WBs/7/Fig7-7A-FOXQ1ú¿ó+HCT8+shNC+inhibit NCú+ó+HCT8+shFOXQ1+inhibitor NCú+ó¦HCT8+shNC+ miR-133a-3p-inhibitorú+ó_HCT8+shFOXQ1+ miR-133a-3p-inhibitorú¬.tif]

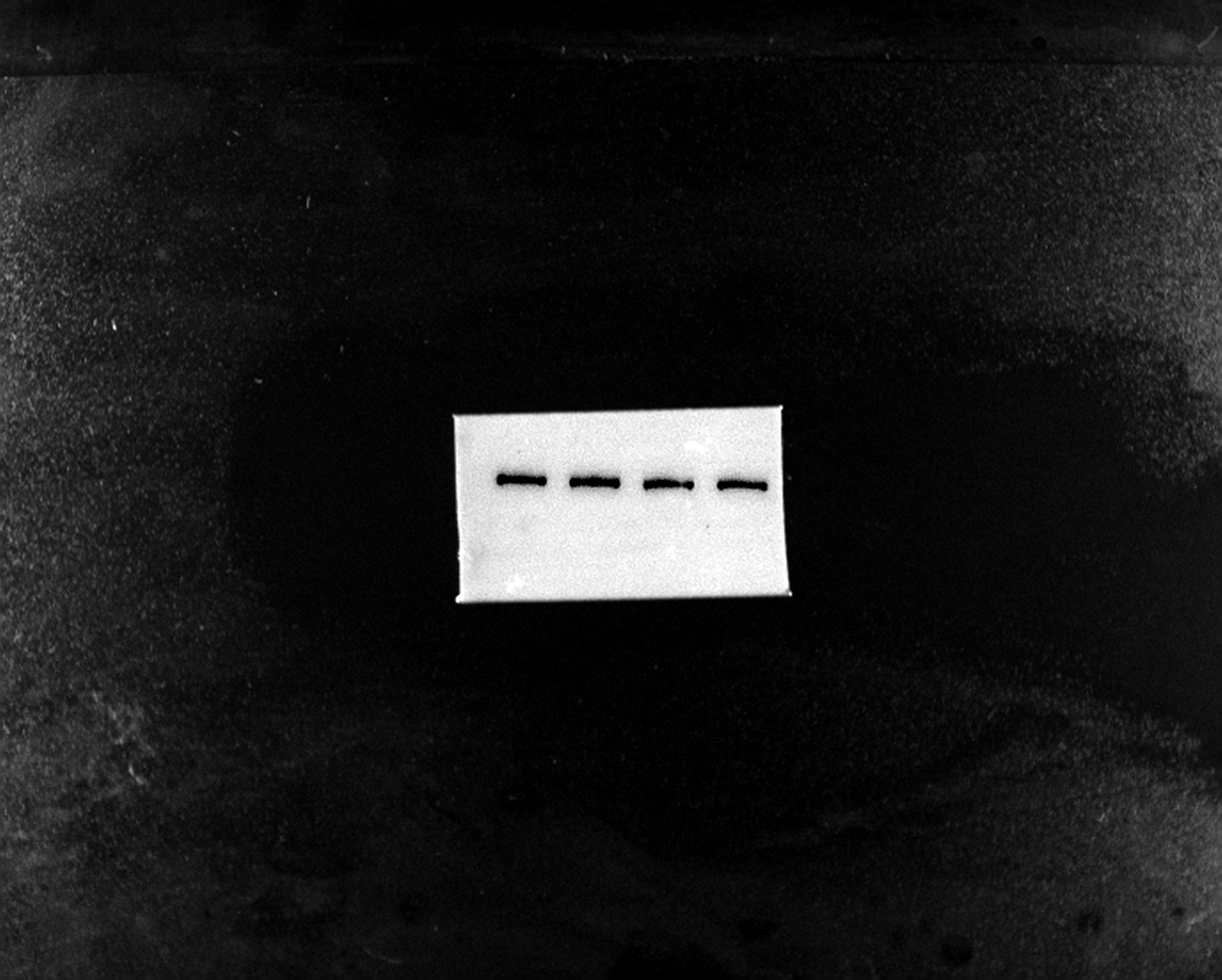

Supplement: Supplementary file 1 [file DataSheet_1.zip › WBs/7/Fig7-7A-GAPDHú¿ó+HCT8+shNC+inhibit NCú+ó+HCT8+shFOXQ1+inhibitor NCú+ó¦HCT8+shNC+ miR-133a-3p-inhibitorú+ó_HCT8+shFOXQ1+ miR-133a-3p-inhibitorú¬.tif]
